# Supplementary material for: Implementation and effectiveness of non-specialist mediated interventions for children with Autism Spectrum Disorder: A systematic review and meta-analysis
Source: PLoS One. 2019 Nov 8;14(11):e0224362. doi: 10.1371/journal.pone.0224362 (PMC6839885; doi:10.1371/journal.pone.0224362)

**Supplementary Online Content**

Supplementary Figure A: Meta-regression analysis for quality of studies

Supplementary Figure B: Meta-regression analysis for duration of intervention program

Supplementary Figure C: Meta-regression analysis for number of sessions of intervention program

Supplementary Figure D: Funnel plot for social skills

Supplementary Figure E: Funnel plot for severity of symptoms

Supplementary Figure F: risk of bias for all studies

**Figure A: Meta-regression analysis for quality of studies**

**Figure B: Meta-regression analysis for duration of intervention program**

**Figure C: Meta-regression analysis for number of sessions of intervention program**

**Figure D: Funnel plot for social skills**

**Figure E: Funnel plot for severity of overall symptoms**

**Figure F: risk of bias for all studies**


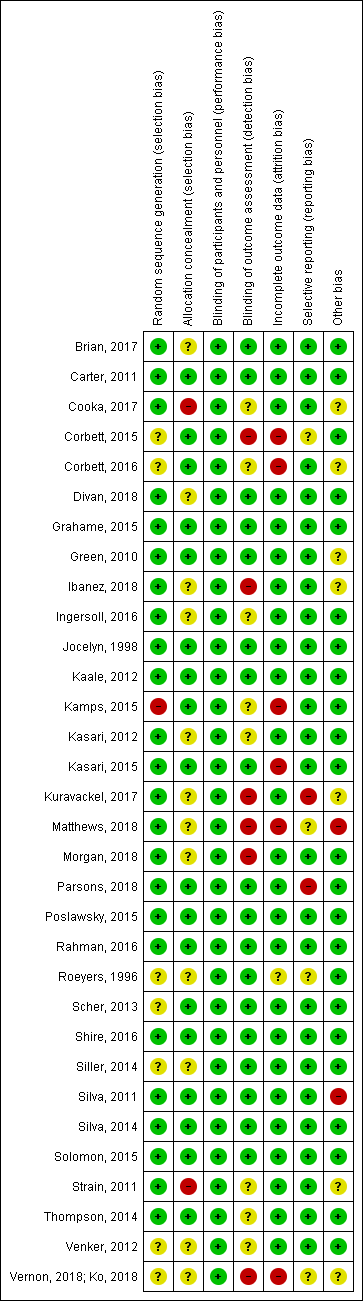

Supplement: S4 File — This file has following figures: a) Meta-regression analysis for quality of studies b) Meta-regression analysis for duration of intervention program c) Meta-regression analysis for number of sessions of intervention program d) Funnel plot for social skills e) Funnel plot for severity of symptoms f) Risk of bias for all studies. (DOCX) [file pone.0224362.s004.docx]
